# Machine learning prediction of survival in centenarians after age 100: a retrospective, population-based cohort study

**DOI:** 10.1093/gerona/glaf218

**Published:** 2025-10-09

**Authors:** Jonathan K L Mak, Noel C Yue, Gloria Hoi-Yee Li, Jacqueline K Yuen, Tung Wai Auyeung, Kathryn Choon Beng Tan, Ching-Lung Cheung

**Affiliations:** Department of Pharmacology and Pharmacy, Li Ka Shing Faculty of Medicine, The University of Hong Kong, Hong Kong SAR, China; Department of Medical Epidemiology and Biostatistics, Karolinska Institutet, Stockholm, Sweden; Department of Pharmacology and Pharmacy, Li Ka Shing Faculty of Medicine, The University of Hong Kong, Hong Kong SAR, China; Department of Health Technology and Informatics, The Hong Kong Polytechnic University, Hong Kong SAR, China; Department of Medicine, School of Clinical Medicine, Li Ka Shing Faculty of Medicine, The University of Hong Kong, Hong Kong SAR, China; Division of Behavioural Sciences, School of Public Health, Li Ka Shing Faculty of Medicine, The University of Hong Kong, Hong Kong SAR, China; Jockey Club Institute of Ageing, The Chinese University of Hong Kong, Hong Kong SAR, China; Department of Medicine, School of Clinical Medicine, Li Ka Shing Faculty of Medicine, The University of Hong Kong, Hong Kong SAR, China; Department of Pharmacology and Pharmacy, Li Ka Shing Faculty of Medicine, The University of Hong Kong, Hong Kong SAR, China; Hinda and Arthur Marcus Institute for Aging Research, Hebrew SeniorLife, Boston, Massachusetts, United States

**Keywords:** Centenarians, Machine learning, Prediction model, Mortality, Oldest-old

## Abstract

**Background:**

Whether survival at extreme ages can be accurately predicted remains unclear. This study explored the feasibility of using machine learning (ML) and electronic health records (EHRs) to predict mortality in centenarians and identify key survival determinants.

**Methods:**

We analyzed 9718 centenarians (83% women) from the population-based EHR database in Hong Kong (2004-2018). Data were randomly split into 70% training and 30% testing cohorts. Using 82 predictors, including demographics, diagnoses, prescriptions, and laboratory results, we trained stepwise logistic regression and four ML algorithms to predict 1-year, 2-year, and 5-year all-cause mortality after age 100. Model performance was evaluated using discrimination (area under the receiver operating characteristic curve [AUROC]) and calibration metrics. In an independent cohort of 174 606 oldest-old adults aged 85-105 years, we further compared AUROCs of models incorporating the identified predictors versus comorbidity and frailty scores across different age groups.

**Results:**

Among the ML models, eXtreme Gradient Boosting algorithm provided the best performance, with AUROCs of 0.707 (95% CI = 0.685-0.730) for 1-year mortality and 0.704 (0.686-0.723) for 2-year mortality in the testing cohort. However, all models showed poor calibration for 5-year mortality. Top three predictors of mortality included lower albumin levels, more frequent hospitalizations, and higher urea levels. Models including these predictors consistently outperformed comorbidity and frailty for mortality prediction among oldest-old adults.

**Conclusions:**

Utilizing ML models and routinely collected EHRs can predict short-term survival in centenarians with moderate accuracy. Further research is needed to determine whether mortality predictors differ across age in the oldest-old population.

## Introduction

As life expectancy continues to rise, more individuals are expected to reach an extreme old age in the coming years. Globally, the number of centenarians, that is, those aged 100 years or older, has doubled from 220 000 in 2005 to 451 000 in 2015 and is projected to surpass 25 million by 2100.^1^ Compared to individuals with shorter lifespans, centenarians are a select group who typically delay or avoid the onset of major age-related diseases such as cancers, heart diseases, and dementia.^2–4^ Notably, centenarians are not a homogeneous group; they exhibit distinct health profiles in terms of diseases,^5^^,^^6^ cognitive and physical function,^7^^,^^8^ and healthcare utilization.^9^^,^^10^ While over half of centenarians die within 2 years of reaching age 100,^10^^,^^11^ some live beyond age 105 (“semi-supercentenarians”) or even 110 (“supercentenarians”). Understanding factors influencing survival beyond age 100 can provide insights into healthy longevity. Moreover, accurately predicting mortality among centenarians can guide clinical decisions and facilitate end-of-life care planning that aligns with patients’ preferences.^12^

Predictors of survival in the oldest-old remain poorly studied. Conventional predictors in younger populations, such as socioeconomic status, smoking, chronic diseases, and genetics, are less predictive of mortality in the oldest-old.^13–15^ It has been suggested that predictors of mortality in centenarians may differ from those observed in younger-old populations,^14^ and whether survival at extreme ages can be accurately predicted remains largely unknown. Although some studies have highlighted cognitive and physical functions as the key predictors of survival beyond age 100,^16–19^ performing such assessments in centenarians can sometimes be challenging due to high prevalence of frailty and cognitive impairment.^20^ Thus, development of a prediction algorithm using electronic health record (EHR) is considered to be a pragmatic approach to predict mortality in centenarians. Other than comorbidity and frailty scores, which are commonly used to predict mortality in older adults,^21^^,^^22^ machine learning (ML) models have been increasingly applied to EHR data in recent years to develop prognostic tools in geriatrics^23^ and have shown increased power and accuracy in predicting clinical outcomes.^24^^,^^25^ However, few studies have focused specifically on the oldest-old.^26^ One study in 2005 developed a neural network model to predict 1-year mortality in a sample of 110 centenarians.^27^ While this highlights the potential of ML models for mortality prediction in centenarians, the study had a small sample size and did not formally assess the accuracy of the prediction model.^27^ We hypothesized that applying ML models to population-based EHRs could improve mortality prediction at extreme ages and uncover new predictors of longevity.

To this end, we leveraged the population-based EHR data from Hong Kong, a city with the world’s highest life expectancy,^28^ to develop and validate ML-based mortality prediction models for centenarians, the group of individuals who live to the most extreme ages. Similar to other long-lived populations, Hong Kong is experiencing a surge in centenarians and an increasing healthcare burden.^29^ Our primary aim was to investigate the feasibility of utilizing ML models on routinely collected EHR data, including diagnoses, prescriptions, admissions, and laboratory results, to stratify centenarians by short-term (1-year, 2-year, and 5-year) mortality risk and to identify the main determinants of survival at extreme ages. To evaluate if our findings may be applicable to the broader oldest-old population, we further compared discrimination performance of models incorporating our identified predictors versus existing comorbidity and frailty scores^21^^,^^22^ in an independent cohort of oldest-old adults aged 85-105 years.

## Methods

### Study population

We conducted a retrospective cohort study using the territory-wide EHR database in Hong Kong—the Clinical Data Analysis and Reporting System (CDARS). The CDARS is managed by the Hong Kong Hospital Authority, the public healthcare service provider that manages 43 hospitals and institutions and 123 outpatient clinics, serving >80% of all hospital admissions in Hong Kong.^30^ The EHRs in the CDARS, available since 1993, include demographics, disease diagnoses (International Classification of Disease, 9th revision [ICD-9] codes), drug prescriptions (British National Formulary [BNF]), admissions, and laboratory test results. The accuracy of diagnosis codes in the CDARS has been validated for various diseases.^31–33^

A flowchart of sample selection is provided in Figure S1 (see online supplementary material for a color version of this figure). Initially, 12 862 individuals aged ≥100 years were identified from the CDARS, who had at least one admission record at in-patient, out-patient, or accident and emergency services between January 1, 2004, and December 31, 2018. Subsequently, we excluded those lost to follow-up or those who turned 100 before the year 2000 (due to limited baseline data availability), resulting in a total of 9718 centenarians in the analysis. Centenarians were followed from the date they reached 100 years (index date) until the date of death or end of study (December 31, 2023; ie, a minimum of 5-year follow-up), whichever came first.

To examine the generalizability of our findings to the oldest-old population, we identified a cohort of oldest-old adults aged 85-105 years who had at least one clinical record in the CDARS during January 1 to December 31, 2019 (*n* = 174 606). Index date was defined as January 1, 2019, and individuals were followed from index date until date of death or end of the 5-year follow-up period.

This study was approved by the Institutional Review Board of The University of Hong Kong/Hospital Authority Hong Kong West Cluster (Ref.: UW 24-347). As the study only involved anonymized EHR data, the need for informed consent is waived in accordance with relevant regulations in Hong Kong.

### Predictor variables

From the CDARS, we initially identified 425 potential predictors, including sex (indicator), number of prior hospitalizations (count), 301 previously validated disease phenotypes (binary),^34^ 105 drug prescription items categorized by BNF chapters (binary), and 17 common laboratory test items with a missingness rate of <30% (continuous). Data were primarily retrieved within 1 year prior to index date. To minimize missing information, we used a 5-year look-back period to capture disease diagnoses as they may not have been repeatedly coded in the CDARS. For laboratory items, the most recent record within 5 years before index date, if available, was used. Missing values for the remaining laboratory items were imputed with the median values. As a sensitivity analysis, we repeated the analysis using k-nearest neighbor imputation, but the model performance was essentially unchanged. Variables with zero or near-zero variance, defined as a percentage of unique values less than 10% or a frequency ratio (frequency of most common to second most common values) greater than 95:5,^35^ were excluded. Consequently, a total of 82 variables (features) were included in the analysis (Table S1).

### Outcomes

We assessed 1-year, 2-year, and 5-year all-cause mortality from the date of reaching age 100 years (index date). Information on date and causes of death (ICD-10) was obtained from the CDARS and the Hong Kong Death Registry.

### Statistical analysis

#### Development of machine learning models

The study population was randomly split into a 70% training cohort for developing ML algorithms (*n* = 6803) and a 30% testing cohort for evaluating model performance (*n* = 2915).

Using the R package “caret,”^35^ we trained five algorithms to predict 1-, 2-, and 5-year mortality after age 100 in the training cohort. Using a conventional statistical method as the baseline reference, we first developed a logistic regression (LR) model, which also incorporates a stepwise variable selection process that identifies the most informative predictors guided by the lowest Akaike Information Criteria. Based on the selected variables, four additional ML models were then derived, including neural network with a single hidden layer, random forest, gradient boosting machine (GBM), and eXtreme Gradient Boosting (XGBoost). Neural network is a highly flexible model that can learn complex patterns in the data and capture non-linear relationships.^36^ Random forest is an ensemble learning method that constructs multiple decision trees to mitigate overfitting.^37^ As another ensemble learning method, both GBM and XGBoost use gradient boosting to iteratively refine predictions, which provide an optimal balance between training speed and predictive accuracy.^38^ Hyperparameters for the neural network, random forest, GBM, and XGBoost algorithms were optimized with 10 rounds of 10-fold cross-validation in the training cohort to maximize the area under the receiver operating characteristic curve (AUROC). The final selected hyperparameters are listed in Table S2.

#### Evaluation of model performance

To determine the best algorithm for mortality prediction, we assessed both discrimination and calibration performance in the testing cohort. Discrimination was assessed using the AUROC,^39^ which evaluates the model’s ability to distinguish between individuals with and without the mortality outcome. DeLong’s tests were used to compare the AUROCs of the ML algorithms against the LR model (reference model). Calibration plots were used to visually inspect how well the predicted probabilities of mortality aligned with observed outcomes. A calibration intercept close to 0 and a slope close to 1 indicate good calibration.^39^ The Brier score was calculated as the mean squared error between the actual outcome and predicted probability, where a lower score indicates a higher overall accuracy.^39^ Other performance metrics, including sensitivity, specificity, positive predictive value (PPV; ie, precision), negative predictive value (NPV), *F*1 score, and accuracy were also examined.

To identify the most important predictors for mortality, we ranked the variable importance based on Shapley additive explanations (SHAP) values, which quantifies the contribution of each feature to the model’s predictions, thereby increases interpretability of ML models.^40^ A positive SHAP value indicates an increased mortality risk, and vice versa.

We compared the discrimination performance of the best-performing ML models with a reduced LR model including only the top three predictors (as determined by SHAP) and sex. As comorbidity and frailty are commonly used to predict mortality in the general older population,^41^^,^^42^ we also compared our developed models with LR models containing sex plus either the Charlson comorbidity index (CCI; based on 17 comorbidities)^43^ or the Hospital Frailty Risk Score (HFRS; derived from 109 frailty-related diagnoses)^21^ to test if our identified predictors may outperform existing comorbidity or frailty scores.

Considering potential inaccuracies in recorded birth years among those with an exceptionally old age, we performed a sensitivity analysis limiting to those died before age 110 years in the testing cohort (*n* = 2831) to assess the robustness of model performance.

Finally, in the independent cohort of oldest-old adults, we compared discrimination of four LR models for 1-, 2-, and 5-year mortality: (1) age and sex; (2) age, sex, and comorbidity (CCI); (3) age, sex, and frailty (HFRS); and (4) age, sex, and the top three predictors identified from our ML models. Analyses were stratified by 5-year age groups (85-89, 90-94, 95-99, and 100-105 years).

All analyses were conducted using R version 4.3.2 (R Project for Statistical Computing). Model development and preprocessing were performed using R package “caret” (version 7.0-1) (Appendix); calibration was assessed using “rms” (v7.0-0); AUROCs were computed using “pROC” (v1.18.5); and plots were generated using “ggplot2” (v3.5.1). A 2-sided *p *< .05 was considered statistically significant.

## Results

### Characteristics of Hong Kong centenarians

As shown in Figure S2 (see online supplementary material for a color version of this figure), the total number of Hong Kong centenarians within the CDARS has increased more than 3-fold from 783 women and 99 men in 2004 to 2432 women and 508 men in 2018. Number of deaths among centenarians also increased from 217 women and 32 men in 2004 to 636 women and 140 men in 2018, although the death rate remained similar over time.

Our main analysis included 9718 centenarians (83.0% women), with 6803 in the training cohort and 2915 in the testing cohort (Table 1). The median age at death was 102.7 years (Figure S3, see online supplementary material for a color version of this figure), with 25.6%, 45.5%, and 82.6% died within 1, 2, and 5 years after reaching age 100, respectively. The most common cause of death was pneumonia (46.9%), followed by heart diseases (9.0%) and malignant neoplasms (3.1%). Descriptive statistics of all the 82 potential predictors are presented in Table S3, which shows that centenarians who died within a year after age 100 were more likely to be males, had more hospitalizations, and generally had a higher prevalence of baseline diseases and drug prescriptions.

**Table 1.** Characteristics of centenarians included in the training and testing cohorts.

Characteristic	Total (*n* = 9718)	Training (*n* = 6803)	Testing (*n* = 2915)	*p*
**Sex, *n* (%)**				.448
** Women**	8069 (83.0)	5662 (83.2)	2407 (82.6)	
** Men**	1649 (17.0)	1141 (16.8)	508 (17.4)	
**Year of birth, *n* (%)**				.081
** 1900-1904**	882 (9.1)	593 (8.7)	289 (9.9)	
** 1905-1909**	2008 (20.7)	1443 (21.2)	565 (19.4)	
** 1910-1914**	3297 (33.9)	2307 (33.9)	990 (34.0)	
** 1915-1918**	3531 (36.3)	2460 (36.2)	1071 (36.7)	
**Age at death, mean (SD)**	102.7 (2.2)	102.7 (2.2)	102.7 (2.2)	.220
**1-year mortality after age 100, *n* (%)**	2492 (25.6)	1783 (26.2)	709 (24.3)	.054
**2-year mortality after age 100, *n* (%)**	4418 (45.5)	3097 (45.5)	1321 (45.3)	.869
**5-year mortality after age 100, *n* (%)**	8028 (82.6)	5651 (83.1)	2377 (81.5)	.074
**Main cause of death^a^, *n* (%)**				.486
** Pneumonia**	4560 (46.9)	3183 (46.8)	1377 (47.2)	
** Diseases of heart**	877 (9.0)	620 (9.1)	257 (8.8)	
** Malignant neoplasms**	305 (3.1)	217 (3.2)	88 (3.0)	
** Septicemia**	283 (2.9)	212 (3.1)	71 (2.4)	
** Cerebrovascular diseases**	263 (2.7)	197 (2.9)	66 (2.3)	
** Nephritis, nephrotic syndrome and nephrosis**	181 (1.9)	122 (1.8)	59 (2.0)	
** Dementia**	173 (1.8)	110 (1.6)	63 (2.2)	
** Chronic lower respiratory diseases**	80 (0.8)	56 (0.8)	24 (0.8)	
** COVID-19**	43 (0.4)	28 (0.4)	15 (0.5)	
** Diabetes**	7 (0.1)	5 (0.1)	2 (0.1)	
** All other causes**	2674 (27.5)	1862 (27.4)	812 (27.9)	
** Unknown**	40 (0.4)	29 (0.4)	11 (0.4)	
** Alive during follow-up^b^**	232 (2.4)	162 (2.4)	70 (2.4)	

Abbreviations: COVID-19, coronavirus disease 2019; SD, standard deviation.

a Categorized using ICD-10 codes for the leading causes of death defined by the Department of Health, Government of the Hong Kong SAR: pneumonia (J12-J18); diseases of heart (I00-I09, I11, I13, I20-I51); malignant neoplasms (C00-C97); septicemia (A40-A41); cerebrovascular diseases (I60-I69); nephritis, nephrotic syndrome and nephrosis (N00-N07, N17-N19, N25-N27); dementia (F01-F03); chronic lower respiratory diseases (J40-J47); COVID-19 (U07); diabetes (E10-E14).

b Individuals were followed from their date of reaching age 100 (index date) to their age at death or end of follow-up (December 31, 2023).

### Comparison of machine learning algorithms

Performance of the LR model and the four ML algorithms is detailed in Table 2 and Table S4. For 1-year mortality prediction, the XGBoost model exhibited the highest AUROC in the testing cohort (0.707; 95% CI = 0.685-0.730), closely followed by random forest (0.707; 0.684-0.730) and GBM (0.706; 0.683-0.728) (Table 2). However, DeLong’s test indicated that the AUROCs of all ML models were not significantly different from the LR model. Both XGBoost and GBM models demonstrated good calibration, with slopes close to 1 and intercepts close to 0 (Figure S4, see online supplementary material for a color version of this figure); they also had the lowest Brier score (0.163) (Table 2). All ML models had an accuracy >0.77, an *F*1 score >0.86, and had high sensitivity (ranging from 0.781 to 0.794) and PPV (0.945-0.971) but relatively low specificity (0.578-0.626).

**Table 2.** Model performance in predicting 1-year and 2-year mortality among centenarians in training and testing cohorts.

Model performance	Stepwise LR	Neural network	Random forest	Gradient boosting machine	XGBoost
**1-year mortality**					
**Training cohort**					
** AUROC (95% CI)**	0.711 (0.697-0.725)	0.717 (0.703-0.731)	0.936 (0.929-0.944)	0.723 (0.709-0.737)	0.729 (0.715-0.743)
**Testing cohort**					
** AUROC (95% CI)**	0.704 (0.681-0.726)	0.701 (0.678-0.724)	0.707 (0.684-0.730)	0.706 (0.683-0.728)	0.707 (0.685-0.730)
***p*-value (DeLong)**	Reference	0.299	0.578	0.606	0.357
** Brier score**	0.163	0.164	0.164	0.163	0.163
** Sensitivity**	0.796	0.794	0.781	0.794	0.794
** Specificity**	0.595	0.578	0.626	0.587	0.592
** PPV**	0.946	0.945	0.971	0.947	0.948
** NPV**	0.247	0.236	0.151	0.233	0.236
***F*1 score**	0.865	0.863	0.866	0.864	0.864
** Accuracy**	0.776	0.772	0.772	0.774	0.775
**2-year mortality**					
**Training cohort**					
** AUROC (95% CI)**	0.705 (0.693-0.717)	0.717 (0.705-0.730)	0.959 (0.955-0.964)	0.723 (0.711-0.735)	0.722 (0.710-0.735)
**Testing cohort**					
** AUROC (95% CI)**	0.697 (0.678-0.716)	0.697 (0.678-0.716)	0.703 (0.684-0.722)	0.704 (0.685-0.723)	0.704 (0.686-0.723)
***p*-value (DeLong)**	Reference	0.999	0.291	0.088	0.068
** Brier score**	0.217	0.218	0.219	0.216	0.216
** Sensitivity**	0.654	0.659	0.656	0.660	0.658
** Specificity**	0.655	0.665	0.645	0.653	0.655
** PPV**	0.781	0.789	0.765	0.772	0.775
** NPV**	0.501	0.507	0.516	0.519	0.515
***F*1 score**	0.712	0.718	0.706	0.711	0.712
** Accuracy**	0.654	0.661	0.652	0.657	0.657

Abbreviations: AUROC, area under the receiver operating characteristic curve; CI, confidence interval; LR, logistic regression; NPV, negative predictive value; PPV, positive predictive value; XGBoost, eXtreme Gradient Boosting.

Similar results were found for 2-year mortality, where the XGBoost model had the highest AUROC (0.704; 95% CI = 0.686-0.723) and lowest Brier score (0.216) (Table 2) and was well-calibrated in the testing cohort (Figure S5, see online supplementary material for a color version of this figure). However, none of the ML models had good predictive performance for 5-year mortality, as suggested by the poor calibration (Figure S6, see online supplementary material for a color version of this figure) as well as low PPV and low *F*1 score (Table S4). Model performance remained essentially unchanged in the sensitivity analysis limiting to individuals aged below 110 years (Table S5).

### Top predictors of mortality after age 100

**Figure 1.**
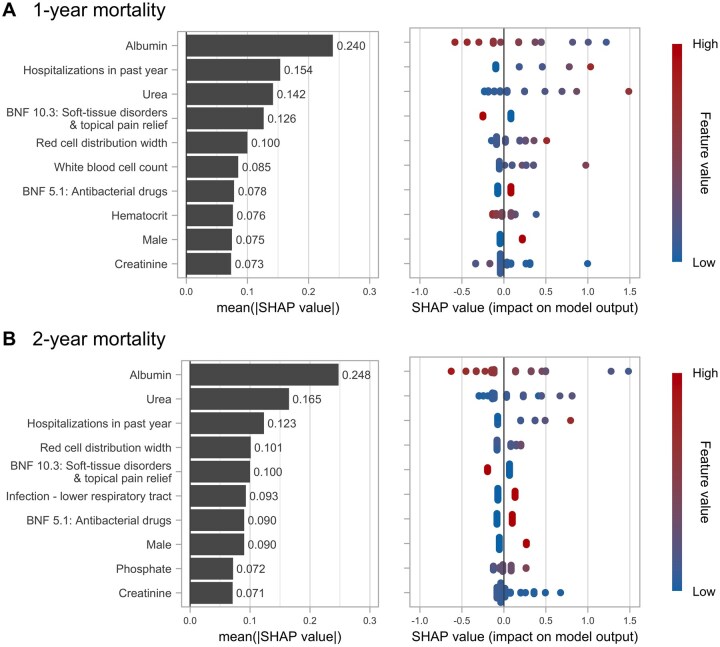
Shapley additive explanations (SHAP) values of variable importance for the XGBoost models in predicting (A) 1-year and (B) 2-year mortality among centenarians. Variables (features) are ranked by their mean absolute SHAP value, from the most important (top) to least (bottom). The left panels provide a global interpretation of feature importance in mortality prediction. In the right panels, each dot represents an individual prediction result. The SHAP values indicate the distribution of the predictions for each feature; a positive value indicates a contribution to mortality whereas a negative value indicates a contribution to survival. The color scheme shows the value of each feature. For example, a higher albumin level is associated with a decreased risk of mortality, and male sex is associated with an increased risk of mortality. XGBoost, eXtreme Gradient Boosting.

As the XGBoost models had the best discrimination and calibration performance, we further identified the most important predictors from these models based on their mean absolute SHAP values (Figure 1). The top predictors that were associated with increased risks of both 1-year and 2-year mortality included lower albumin levels, more frequent hospitalizations in the past year, higher serum urea levels, absence of soft-tissue inflammation medication use (BNF 10.3; including topical non-steroidal anti-inflammatory drugs [NSAIDs] such as methyl salicylate and diclofenac), higher red cell distribution width (RDW), use of antibacterial drugs (BNF 5.1), male sex, and lower creatinine levels (Figure 1).

**Figure 2.**
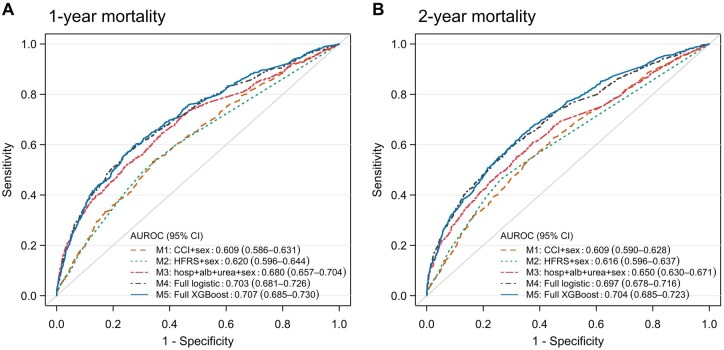
Receiver operating characteristic curves for predicting (A) 1-year and (B) 2-year mortality among centenarians in the testing cohort. The first 4 models are logistic regression models. Model 1 included Charlson comorbidity index (CCI) and sex. Model 2 included Hospital Frailty Risk Score (HFRS) and sex. Model 3 included the top three identified predictors, including hospitalizations, albumin, and urea, as well as sex. Model 4 is the full stepwise logistic regression model. Model 5 is the full XGBoost model. The AUROC of the full XGBoost model was significantly higher than that of the first 3 models in predicting both 1-year and 2-year mortality (all DeLong’s test *p* < .001) but was not significantly different from that of the full logistic regression model (DeLong’s test *p* = .357 for 1-year mortality and *p* = .068 for 2-year mortality). AUROC, area under the receiver operating characteristic curve; CI, confidence interval; XGBoost, eXtreme Gradient Boosting.

To examine if a reduced model may be sufficient to predict mortality in centenarians, we compared the AUROC of a LR model including only top predictors (albumin, prior hospitalizations, urea, and sex) against the full XGBoost and full stepwise LR models in the testing cohort. The reduced model showed lower AUROCs (1-year mortality = 0.680; 2-year mortality = 0.650) than the full models, but outperformed models based on sex plus the CCI (1-year and 2-year mortality = 0.609) or the HFRS (1-year mortality = 0.620; 2-year mortality = 0.616; Figure 2).

### Mortality prediction performance across the oldest-old population

In the independent cohort of 174 606 oldest-old adults, the 1-year mortality rate was 7.9%, 13.3%, 20.4%, and 27.8% for those aged 85-89, 90-94, 95-99, and 100-105 years, respectively. Older age groups had lower albumin, higher urea, more frequent hospitalizations, and higher HFRS and CCI scores (*p *< .05; Table S6). Across all age groups, models incorporating the top identified predictors (model 4: albumin, urea, prior hospitalizations, sex, and age) consistently had higher AUROCs than models based on HFRS or CCI in addition to age and sex (Table 3). However, model discrimination declined with advancing age; for example, the AUROC of model 4 for 1-year mortality was 0.745 (95% CI = 0.739-0.750) in the 85-89 age group, decreasing to 0.717 (0.710-0.724), 0.682 (0.671-0.693), and 0.673 (0.651-0.695) for those aged 90-94, 95-99, and 100-105 years, respectively.

**Table 3.** Comparison of model performance across age groups in the independent cohort of oldest-old adults.

Outcome	Number of individuals^a^	Number of deaths (%)	AUROC (95% CI)^b^			
			Model 1: age + sex	Model 2: age + sex + CCI	Model 3: age + sex + HFRS	Model 4: age + sex + albumin + urea + hospitalizations
**1-year mortality**						
** 85**-**89 years**	106 270	8402 (7.9)	0.585 (0.578-0.591)	0.693 (0.687-0.699)	0.680 (0.674-0.686)	0.745 (0.739-0.750)
** 90**-**94 years**	50 154	6661 (13.3)	0.572 (0.565-0.579)	0.649 (0.642-0.657)	0.653 (0.646-0.661)	0.717 (0.710-0.724)
** 95**-**99 years**	15 326	3121 (20.4)	0.545 (0.534-0.556)	0.605 (0.594-0.616)	0.606 (0.594-0.617)	0.682 (0.671-0.693)
** 100**-**105 years**	2856	795 (27.8)	0.558 (0.535-0.581)	0.601 (0.578-0.624)	0.608 (0.585-0.632)	0.673 (0.651-0.695)
**2-year mortality**						
** 85**-**89 years**	106 270	17 011 (16.0)	0.584 (0.579-0.588)	0.679 (0.675-0.684)	0.681 (0.676-0.685)	0.726 (0.722-0.730)
** 90**-**94 years**	50 154	12 897 (25.7)	0.570 (0.564-0.576)	0.644 (0.638-0.649)	0.652 (0.646-0.657)	0.699 (0.693-0.704)
** 95**-**99 years**	15 326	5732 (37.4)	0.559 (0.549-0.568)	0.614 (0.605-0.623)	0.620 (0.611-0.629)	0.675 (0.666-0.683)
** 100**-**105 years**	2856	1399 (49.0)	0.541 (0.520- 0.562)	0.593 (0.573-0.614)	0.605 (0.585-0.626)	0.654 (0.634-0.674)
**5-year mortality**						
** 85**-**89 years**	106 270	44 720 (42.1)	0.595 (0.591-0.598)	0.668 (0.665-0.672)	0.681 (0.678-0.685)	0.701 (0.697-0.704)
** 90**-**94 years**	50 154	29 901 (59.6)	0.585 (0.580-0.590)	0.654 (0.649-0.659)	0.670 (0.665-0.675)	0.688 (0.684-0.693)
** 95**-**99 years**	15 326	11 688 (76.3)	0.578 (0.568-0.589)	0.632 (0.622-0.642)	0.655 (0.645-0.665)	0.686 (0.676-0.695)
** 100**-**105 years**	2856	2481 (86.9)	0.567 (0.536-0.598)	0.620 (0.590-0.649)	0.647 (0.617-0.676)	0.693 (0.666-0.719)

Abbreviations: AUROC, area under the receiver operating characteristic curve; CCI, Charlson Comorbidity Index; CDARS, Clinical Data Analysis and Reporting System; CI, confidence interval; HFRS, Hospital Frailty Risk Score.

a The cohort included all oldest-old adults aged 85-105 years in 2019 identified from the CDARS (*n* = 174 606). Index date was defined as January 1, 2019. Characteristics of the sample are shown in Table S6.

b All the models were logistic regression models. Model 1 included only age (as of 2019) and sex. Model 2 included age, sex, and comorbidity (CCI). Model 3 included age, sex, and frailty (HFRS). Model 4 included age, sex, and the top three identified predictors in centenarians (albumin, urea, and hospitalizations in past year).

## Discussion

This study leveraged a large, population-based EHR dataset to develop ML models for predicting all-cause mortality in centenarians. Among the ML algorithms, XGBoost provided the best discrimination and calibration for predicting 1-year and 2-year mortality beyond age 100. However, all the models had poor calibration for 5-year mortality. Several key predictors of mortality were identified, including lower albumin levels, more frequent hospitalizations, and higher urea levels. Additionally, in an independent cohort of oldest-old adults, we found that models including these top predictors achieved significantly higher AUROCs for 1-year and 2-year mortality compared to conventional comorbidity and frailty measures across different age groups, indicating their potential use in stratifying short-term mortality risk among centenarians and oldest-old adults in general.

To the best of our knowledge, this is the first study to develop ML models using routinely collected EHR data to predict mortality in centenarians. It was previously hypothesized that survival at such extreme ages may be determined by stochastic factors, as centenarians have already avoided or survived from the major predictors of mortality at younger ages.^14^ However, in a recent study using latent class analysis to group Danish centenarians by their functional and cognitive health, Alvarez et al. showed that there is still a distinct health gradient in which frail centenarians had significantly shorter survival beyond age 100.^16^ Other studies, albeit with relatively small sample sizes, have similarly identified cognitive and physical functioning as factors influencing survival in centenarians,^18^^,^^19^ suggesting that mortality can indeed be predicted even at extreme ages. In line with these observations, our results suggested that an ML model incorporating diagnoses, prescriptions, admissions, and laboratory results data can predict short-term mortality with moderate discrimination and good calibration.

Due to a lack of previous studies on mortality prediction among centenarians or oldest-old adults, we can only compare our results with models developed for older adults in general. Despite having a cohort of older age, our AUROC of ∼0.70 is consistent with previous mortality prediction models for the general older population.^41^^,^^42^ A recent systemic review showed that 67% of the 1-year mortality prediction models developed for community-dwelling older adults aged ≥65 years had similar discrimination levels (AUROCs between 0.70-0.79), with only one model demonstrating excellent performance (0.81).^41^ Likewise, mortality prediction models for older adults living in nursing homes showed a meta-analyzed C-statistics of 0.73.^44^ Among the tested ML algorithms, XGBoost provided the best discrimination and calibration performance, which is an algorithm renowned for its performance with tabular data^45^ and has been applied in mortality prediction across various patient groups.^46–48^ Interestingly, although the XGBoost model achieved the highest AUROCs for both 1- and 2-year mortality, statistical comparison based on DeLong’s test indicated no significant difference compared to the LR model. This is consistent with previous studies suggesting that ML models often do not substantially outperform traditional regression approaches.^24^^,^^49^^,^^50^ This may be due to the underlying relationships between predictors and outcomes being relatively linear and additive, allowing LR to perform nearly as well as the more complex algorithms. The type of predictors, such as the use of structured data and inclusion of primarily binary variables, may also limit the ability of ML algorithms to capture complex nonlinear relationships or interactions.^50^

In our models, albumin consistently ranked as the most important predictor, aligning with existing literature showing a strong association between low serum albumin and increased mortality in the oldest-old.^51^^,^^52^ Albumin, the most abundant plasma protein, is generally considered as a marker of nutrition status in older adults.^53^ Low levels of albumin have been linked to reduced muscle mass and sarcopenia,^54^ which are associated with mortality risk in centenarians.^19^ Albumin is also a negative acute-phase protein,^53^ where a hypoalbuminemia typically reflects an inflammatory state that is caused by infection and certain diseases and could lead to a substantially higher mortality risk in centenarians.^55^ Interestingly, while cardiovascular diseases, cancers, and neurological disorders are strong predictors of mortality in the general older population,^41^ these variables were not among the top predictors in centenarians, possibly due to a low incidence of these diseases among centenarians throughout their lifetime.^4^ Instead, we found that indicators of acute illness such as recurrent hospitalizations and use of antibacterial medications are more closely associated with mortality risk in centenarians. The frequency of hospitalizations reflects overall disease severity and is known to increase mortality risk.^56^ Additionally, our results indicate that pneumonia is the most common cause of death among centenarians in Hong Kong, with recurrent hospitalizations potentially heightening the risk due to the crowded conditions in local hospitals, which may lead to an increased risk of cross-transmission of pneumonia pathogens.^29^ Pneumonia could also be a result of aspiration due to poor physiological functioning that is commonly seen in frail older adults. Moreover, urea was also identified as a top predictor, where elevated serum urea levels typically indicate reduced renal function and dehydration and could lead to higher mortality risk and disease severity in critically ill patients.^57–59^

Our analysis in the independent cohort of oldest-old adults showed that the identified mortality predictors, including albumin, hospitalizations, and urea, are not only pertinent to centenarians but may also be applicable to other age groups in the oldest-old population, as shown by the relatively high discrimination performance of the models in younger subgroups (eg, AUROC for 1-year mortality decreasing from 0.745 in those aged 85-89 to 0.673 in those aged 100-105 years). Importantly, models incorporating these predictors also outperformed the CCI and HFRS in predicting mortality, both are established risk scores and known as strong predictors of mortality in the oldest-old.^60^^,^^61^ Of note, due to limitations in data availability and scope, we did not formally test whether and how mortality predictors may differ across specific age groups. Since the influence of certain factors on mortality risk, such as chronic diseases which centenarians tend to delay or avoid,^4^ as well as genetics,^13^ may change with advancing age, further research is needed to systematically identify the most important predictors of mortality across different age groups in the older population.

Strengths of this study include the use of a comprehensive, population-based EHR database, providing one of the largest datasets for investigating survival determinants in centenarians. As death records were obtained through linkage to the Hong Kong Death Registry, lost to follow-up with unknown death status is unlikely. Although centenarians remain relatively rare, this age group is rapidly expanding globally.^1^ Consistent with previous studies showing an increasing number of centenarian deaths in the Chinese population,^29^^,^^62^ our data showed that both the number of individuals reaching age 100 and centenarians’ death have increased almost 3 times in women and 4 times in men between 2004 and 2018 in Hong Kong. Moreover, recent estimates suggest that Hong Kong people have the highest probability of surviving to age 100 among several long-lived populations, with 12.8% of women and 4.4% of men expected to reach this age.^63^ Therefore, our findings may have profound implications for the world’s aging population, highlighting factors that contribute to increased mortality in centenarians. Our developed ML models can also be potentially integrated into the EHR system to inform end-of-life care planning for centenarians.

Several limitations should also be considered. First, the EHRs in CDARS lacked information on centenarians’ cognitive and functional status, clinical measurements (eg, body mass index, grip strength), lifestyle factors, and socioeconomic variables. While these factors can be proxied through available diagnoses, prescriptions, and laboratory results, incorporating them in future studies may further improve predictive accuracy of the ML models. Second, as the data were anonymized, we were unable to verify the accuracy of the date of birth of centenarians. This may have affected the models’ accuracy, especially for those with a death record at an exceptionally old age (≥110 years). However, sensitivity analysis restricting the sample to individuals below age 110 yielded largely consistent results. Third, we lacked information on healthy centenarians who have never utilized public healthcare services during 2004-2018, as well as those exclusively followed in the private sector. However, we expect this number to be small, since centenarians typically have high healthcare service utilization,^9^^,^^64^ and that the public healthcare system in Hong Kong is highly subsidized and covers ∼80% of the Hong Kong’s older population.^30^ Finally, although the ML models were tested in a holdout testing cohort, the generalizability of our developed models needs to be further examined in other populations. However, centenarians remain relatively rare in many populations; thus, multinational collaboration would be important for future centenarian research.

## Conclusion

Our developed ML models, based on readily available EHR data, offer a means to predict short-term all-cause mortality in centenarians. We identified several key predictors of mortality including lower albumin levels, recurrent hospitalizations, and higher urea levels, and these predictors also seemed to be applicable to other age groups in the oldest-old population. While further studies are needed to validate our findings in diverse populations and determine whether mortality predictors may differ across age in oldest-old adults, these preliminary results suggest that ML models are feasible to identify high-risk individuals even at extreme ages, which may help to facilitate end-of-life care planning for the rapidly growing oldest-old and centenarian population.

## Supplementary Material

glaf218_Supplementary_Data

## Data Availability

This study was conducted using anonymized EHR data from the CDARS database managed by the Hong Kong Hospital Authority (HA). The data cannot be made publicly available due to third-party use restrictions and patient confidentiality concerns. Nevertheless, local academic institutions, government departments, or non-governmental organizations can apply for access to the HA data through the Data Sharing Portal (https://www3.ha.org.hk/data/Provision).
