# Supplementary material for: Machine learning prediction of survival in centenarians after age 100: a retrospective, population-based cohort study
Source: J Gerontol A Biol Sci Med Sci. 2025 Oct 9;80(12):glaf218. doi: 10.1093/gerona/glaf218 (PMC12598932; doi:10.1093/gerona/glaf218)
Supplement: glaf218_Supplementary_Data [file glaf218_supplementary_data.pdf]

Supplementary Material

Supplementary Figure 1. Flowchart of selection of the centenarian cohort. 2

Supplementary Figure 2. Number of Hong Kong centenarians identified from the Clinical Data Analysis and Reporting System (CDARS) between 2004 and 2018. 3

Supplementary Figure 3. Age at death among the included centenarians... 4

Supplementary Figure 4. Calibration plots for 1-year mortality in the testing cohort............................. 5

Supplementary Figure 5. Calibration plots for 2-year mortality in the testing cohort............................. 6

Supplementary Figure 6. Calibration plots for 5-year mortality in the testing cohort............................. 7

Supplementary Table 1. List of the 82 potential predictors used in the machine learning models.......... 8

Supplementary Table 2. Final hyperparameters used in the machine learning models. 13

Supplementary Table 3. Characteristics by death status at 1-year after age 100. 14

Supplementary Table 4. Model performance in predicting 5-year mortality. 16

Supplementary Table 5. Model performance in the sensitivity analysis limiting to individuals died at age <110 years (n=2,831).. 17

Supplementary Table 6. Characteristics of the independent cohort of oldest-old adults. 18

Appendix. R codes for development of machine learning models 19

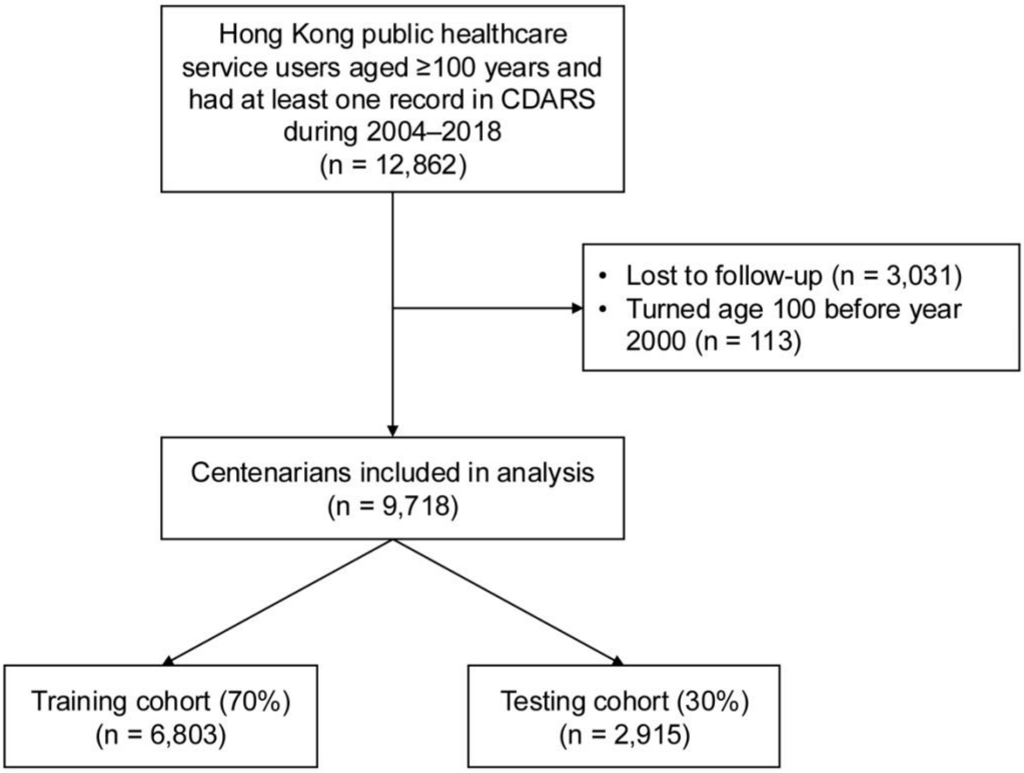

Supplementary Figure 1. Flowchart of selection of the centenarian cohort.

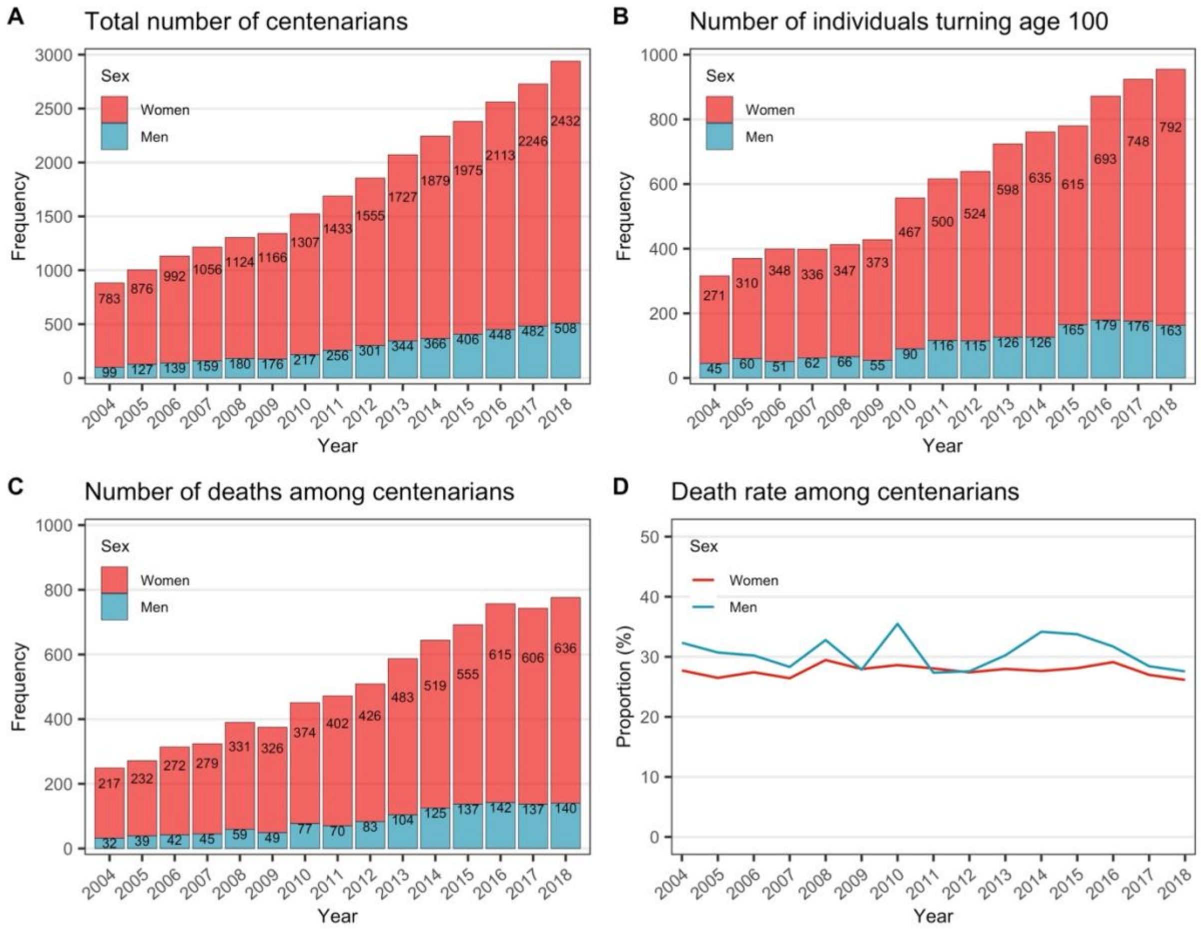

Supplementary Figure 2. Number of Hong Kong centenarians identified from the Clinical Data Analysis and Reporting System (CDARS) between 2004 and 2018.

(A) Total number of centenarians (i.e., prevalent cases); **(B)** number of new centenarians (i.e., incident cases); **(C)** number of deaths among centenarians; **(D)** death rate among centenarians (i.e., proportion of deaths among centenarians).

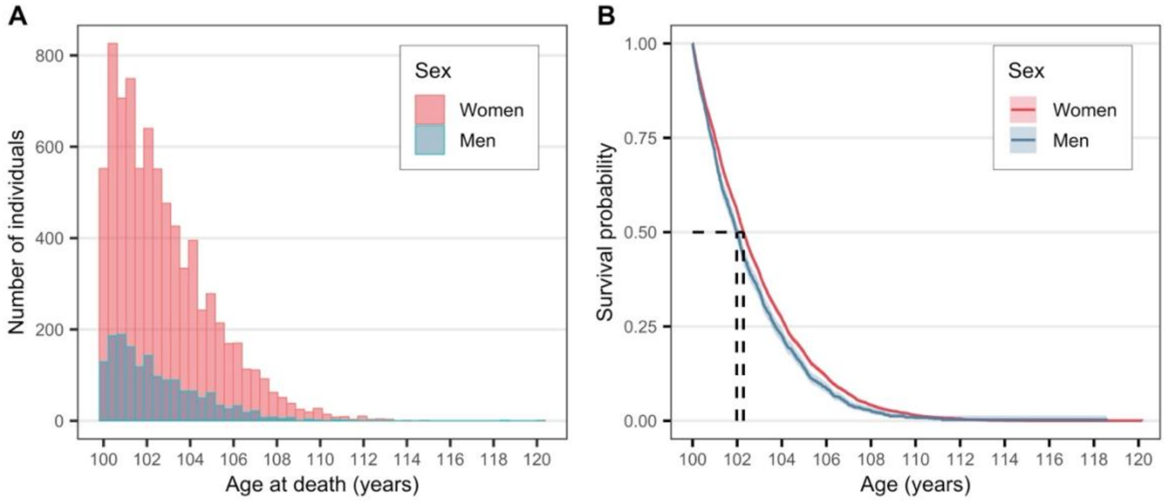

Supplementary Figure 3. Age at death among the included centenarians.

(A) Distribution of centenarians by their age of death, stratified by sex. The mean age at death was 102.7 years in women and 102.4 years in men, maximum age at death was 120.2 years in women and 118.6 in men. **(B)** Kaplan-Meier curves of survival probability among centenarians. The black dotted lines represent median survival, which was 2.3 years in women and 2.0 in men.

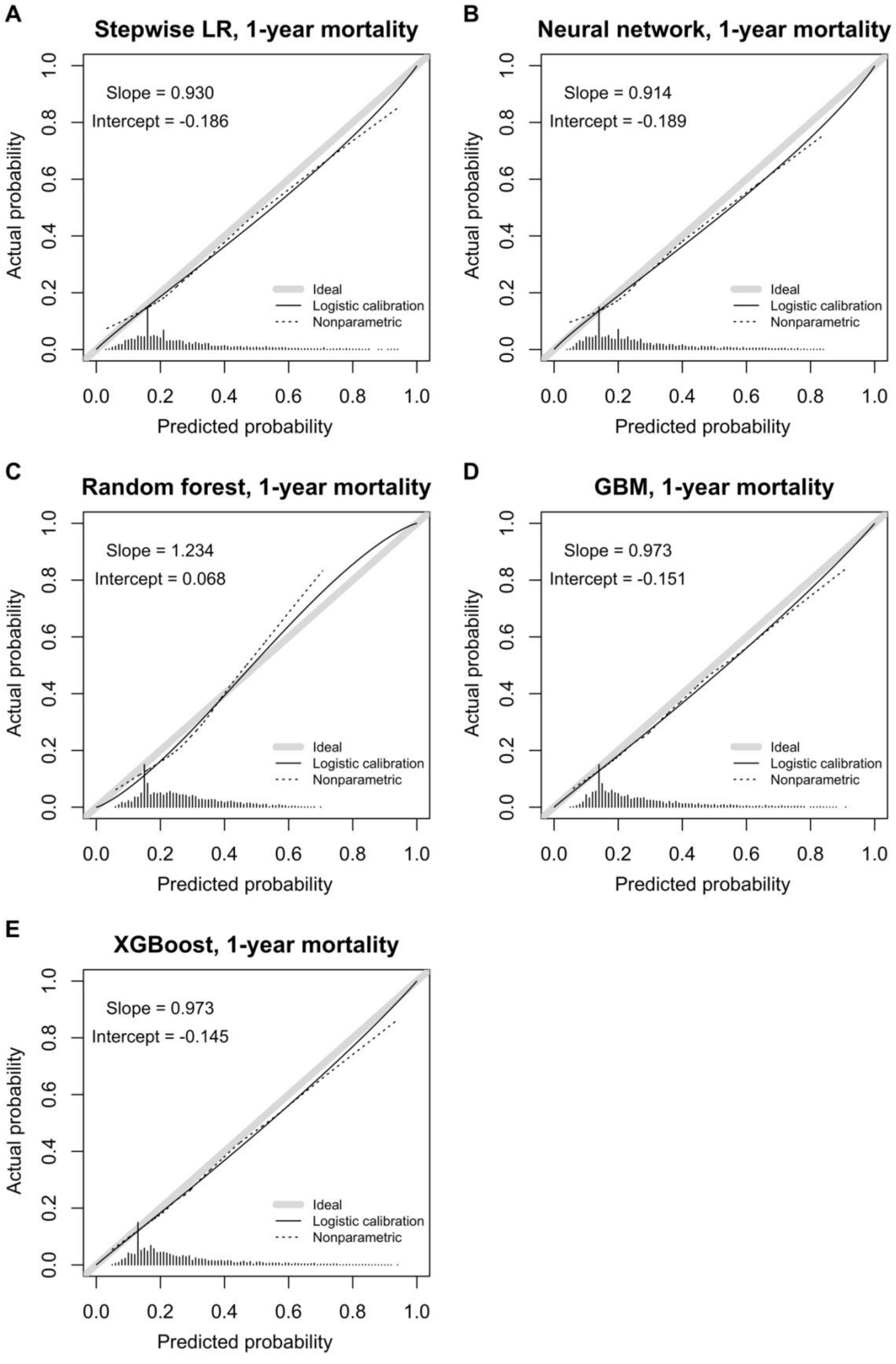

Supplementary Figure 4. Calibration plots for 1-year mortality in the testing cohort.

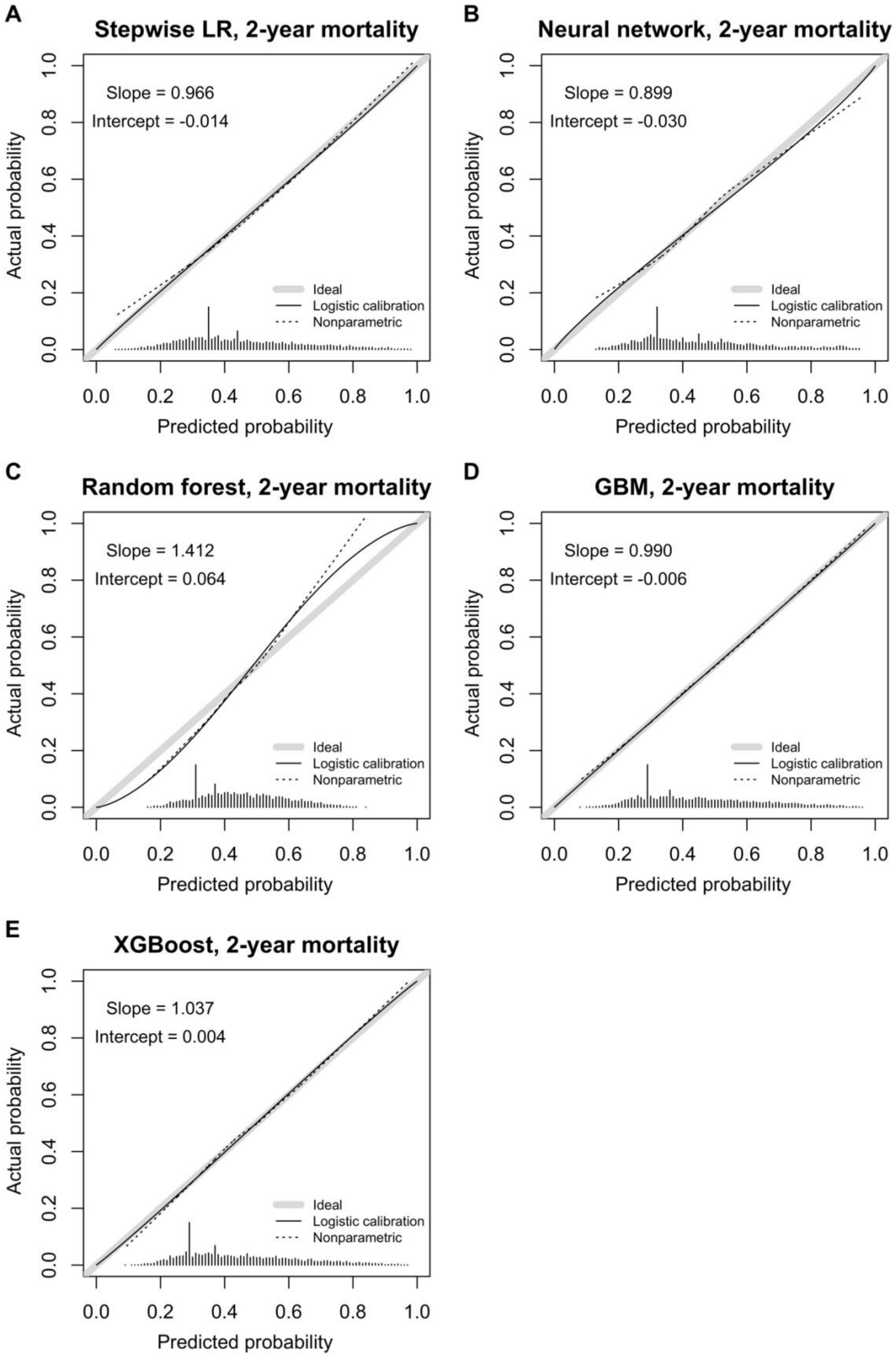

Supplementary Figure 5. Calibration plots for 2-year mortality in the testing cohort.

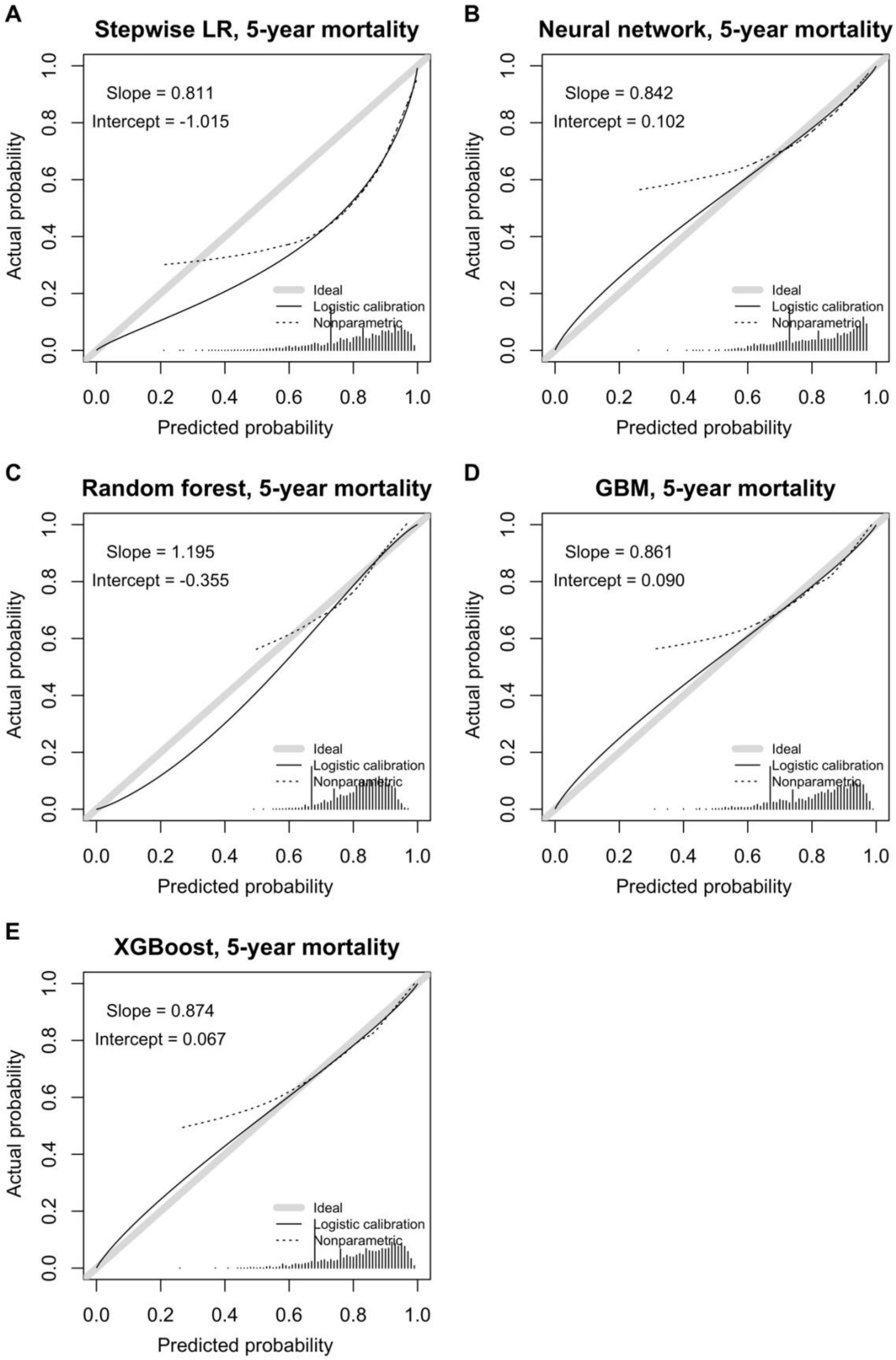

Supplementary Figure 6. Calibration plots for 5-year mortality in the testing cohort.

Supplementary Table 1. List of the 82 potential predictors used in the machine learning models.

No.	Variable	Description
Demographics		
1	Male	Male sex (0=female, 1=male)
2	Hospitalizations	No. of hospitalizations in past year (count)
Disease diagnoses (ICD-9 codes within 5 years before index date; 0=absence of codes, 1=presence)		
3	Alcohol misuse	255.0, 291, 303, 305.0, 349.89, 357.5, 359.4, 425.5, 535.3, 571.0, 571.1, 571.2, 571.3, 577.0, 577.1, V57.89, V65.42
4	Delirium	293, 780.09
5	Dementia	290, 294.1, 294.8, 298.9, 331.0
6	Atrial fibrillation	427.3
7	Coronary heart disease	414.0, 414.1, 414.8, 414.9, 429.2
8	Chronic obstructive pulmonary disease (COPD)	466.0, 490, 491, 492, 493.2, 496
9	Heart failure	398.91, 402.01, 402.11, 402.91, 404.01, 404.03, 404.11, 404.13, 404.91, 404.93, 428
10	Hypertension	401-405
11	Intracerebral hemorrhage	431, 438
12	Ischemic stroke	433.01, 433.11, 433.21, 433.31, 433.81, 433.91, 434.01, 434.11, 434.91, 436, 438
13	Stable angina	411.0, 413.1, 413.9, 786.50
14	Subarachnoid hemorrhage	430, 438
15	Transient ischemic attack (TIA)	362.34, 433, 434, 435.0, 435.1, 435.2, 435.8, 435.9, 437.7
16	Cataract	366.0, 366.1, 366.3, 366.4, 366.5, 366.8, 366.9, 743.30-34
17	Diabetes	250, 357.2, 362.0, 366.41
18	Hip fracture	820
19	Gout	274
20	Osteoarthritis	715, 716
21	Other anemia	281, 284.9, 285, 666.10

Supplementary Table 1. *(continued)*

No.	Variable	Description
22	Bacterial infection	001-005, 008.0, 008.1-5, 020-027, 030-037, 038.0, 038.1-4, 039-042, 073, 076, 077.0, 077.98, 078.3, 078.8, 079.88, 079.98, 080-083, 087, 088.0, 088.81, 090-098, 099.0-2, 099.41, 099.5, 100-104, 139.1, 294.1, 320, 323.9, 376.01, 390-395, 397, 398, 420.9, 466.0, 475, 478.1, 478.21-24, 478.29, 481, 482, 483.0, 483.1, 484.3, 484.5, 510, 517.1, 528.1, 530.84, 565.0, 566, 569.5, 590.2, 590.80, 599.0, 601.0, 601.2, 601.3, 603.1, 604, 614, 615, 616.0, 616.3, 616.8, 616.9, 625.8, 680-682, 684, 685.0, 695.1, 695.81, 704.8, 711.0, 711.4, 711.9, 727.09, 727.3, 727.89, 728.86, 729.4, 730, 730.99, 733.99, 760.2, 770.0, 771, 790.7, 791.9, 879.7, 997.5
23	Infection - ear/upper respiratory tract	017.4, 032.0-3, 034.0, 055.2, 075, 098.6, 099.51, 380.10-16, 380.2, 381.0-4, 382, 383, 461-465, 475, 476, 478.1, 478.21, 478.22, 478.24, 478.29
24	Infection - skin	006.6, 017.00-06, 017.1, 020.1, 021.0, 022.0, 027.0, 027.1, 031.1, 032.85, 035, 051, 054.0, 054.9, 054.73, 054.79, 054.8, 057, 074.0, 074.3, 074.8, 078.0, 078.1, 078.4, 079.89, 085.1-4, 085.9, 110, 111, 117.1, 117.2, 117.5, 117.8, 117.9, 132, 134, 136.8, 380.15, 376.01, 680, 681, 682, 684, 685.0, 686, 695.1, 695.81, 701.1, 771.4, 771.8, 879.7, 999.0
25	Infection - other organs	006.8, 006.9, 017.5, 017.6, 017.70-76, 017.8, 017.9, 018, 020.8, 020.9, 021.8, 021.9, 022.8, 022.9, 023-027, 030, 031.8, 031.9, 032.2, 032.8, 036.1, 036.8, 036.9, 040-44, 060, 061, 065, 066, 072.7, 072.9, 073, 074, 079, 080-084, 085.0, 085.5, 085.9, 086.0-2, 088, 090, 091.0, 091.4, 091.5, 091.6, 091.7, 091.8, 091.9, 095, 096, 097, 098.0, 098.81-86, 098.89, 098.9, 099.4, 099.50, 099.59, 099.8, 099.9, 100-104, 112.0, 112.1, 112.5, 112.8, 114.1, 114.3, 114.9, 115.0, 115.1, 115.9, 116.0, 116.1, 116.2, 117, 118, 120, 121, 122.2, 122.3, 122.4, 122.6, 122.7, 122.9, 123, 124, 125, 126, 127.0, 127.2, 127.3, 127.4, 128, 130.3, 130.7, 130.8, 130.9, 131.01, 131.8, 131.9, 133, 134.0, 134.2, 136, 137.0, 137.4, 139.8, 275.4, 294.1, 321.1, 322.1, 336.8, 354.9, 356.8, 372.15, 372.30, 373.6, 376.13, 476, 482.83, 484, 487.1, 487.8, 513.1, 528.1, 711.00, 711.4, 711.5, 711.6, 711.7, 711.8, 716.80, 727.09, 727.3, 727.89, 728.0, 728.86, 728.89, 729.4, 771.0, 771.1, 771.2, 771.5, 771.6-8, 781.9, 789.2, 790.7, 790.8, V09
26	Infection - digestive system	001, 002, 003.0, 004, 005, 006.0, 006.1, 006.2, 006.8, 006.9, 007-009, 014, 021.1, 022.2, 055.79, 078.82, 127, 129, 136.5, 535.40, 558.9, 569.5, 787.91
27	Lower respiratory tract infection	006.4, 010, 011, 012, 017.90, 018.80, 020.3, 020.4, 020.5, 021.2, 022.1, 031.0, 033, 039.1, 052.1, 055.1, 078.5, 112.4, 114.0, 114.4, 114.5, 115.05, 117.1, 117.3, 117.5, 122.1, 130.4, 136.3, 466, 480-483, 484.1, 484.3, 484.5, 484.7, 484.8, 485, 486, 487.0, 491.21, 496, 505, 510, 513.0, 514, 516.8, 519.8, 530.84, 674.80, 770.0, 770.8

Supplementary Table 1. *(continued)*

No.	Variable	Description
28	Infection - other organisms	008.49, 008.8, 009, 038.8, 038.9, 040.81, 046.0, 046.1, 046.8, 046.9, 099.3, 099.4, 099.8, 099.9, 136.9, 139.8, 290.10, 290.12, 290.13, 321.8, 323.1, 323.2, 323.4, 323.6, 323.8, 323.9, 324, 325, 349.89, 354.9, 356.8, 370.32, 372.0-3, 372.61, 379.99, 380.1, 380.2, 381.0, 381.1, 382, 383, 384.00, 384.01, 384.09, 384.1, 420.90, 421.0, 422.91, 422.92, 425.7, 460-466, 481, 483.8, 484, 485, 486, 491.21, 496, 513, 514, 516.8, 519.8, 558.9, 572.0, 573.1, 573.2, 580.89, 590.1, 595.0, 595.8, 595.9, 597, 607.1, 648.91, 648.93, 674.80, 686.0, 686.8, 686.9, 701.1, 704.8, 711.00, 711.1, 711.3, 711.7, 711.8, 716.8, 720.9, 722.9, 728.0, 728.89, 730.98, 770.0, 770.8, 771, 781.9, 787.91, 997.2, V09
29	Sepsis	003.1, 020.2, 022.3, 027.0, 027.1, 036.2, 036.3, 038, 039.9, 112.5, 648.91, 648.93, 760.2, 771
30	Urinary tract infection (UTI)	099.40, 580.89, 590.1, 590.2, 590.80, 595.0, 595.9, 597, 599.0, 791.9, 997.5
31	Viral infection	008.6, 008.8, 009.0, 042, 045, 046.2, 046.3, 047-049, 051-057, 060-066, 070-072, 074, 075, 077, 078, 079, 088, 138, 139.0, 139.8, 294.1, 321.2, 323.0, 336.8, 344.12, 429.89, 466.0, 466.1, 480, 484.1, 487, 535.40, 570, 711.5, 730.7, 770.0, 771.0, 771.1, 771.2, 771.8, 790.8, 999.0
Drug prescription (BNF within 1 year before index date; 0=absence of drug prescription, 1=presence)		
32	Dyspepsia and gastro-esophageal reflux disease	BNF 1.1
33	Antisecretory drugs and mucosal protectants	BNF 1.3
34	Laxatives	BNF 1.6
35	Diuretics	BNF 2.2
36	Beta-adrenoceptor blocking drugs	BNF 2.4
37	Hypertension and heart failure	BNF 2.5
38	Nitrates, calcium-channel blockers & other antianginal drugs	BNF 2.6
39	Antiplatelet drugs	BNF 2.9
40	Lipid-regulating drugs	BNF 2.12
41	Bronchodilators	BNF 3.1
42	Antihistamines, hypersensitization and allergic emergencies	BNF 3.4
43	Mucolytics	BNF 3.7
44	Cough preparations	BNF 3.9
45	Hypnotics and anxiolytics	BNF 4.1

Supplementary Table 1. *(continued)*

No.	Variable	Description
46	Drugs used in psychoses and related disorders	BNF 4.2
47	Antidepressant drugs	BNF 4.3
48	Drugs used in nausea and vertigo	BNF 4.6
49	Analgesics	BNF 4.7
50	Antibacterial drugs	BNF 5.1
51	Drugs used in diabetes	BNF 6.1
52	Corticosteroids (endocrine)	BNF 6.3
53	Drugs for genitourinary disorders	BNF 7.4
54	Anemias and some other blood disorders	BNF 9.1
55	Fluids and electrolytes	BNF 9.2
56	Minerals	BNF 9.5
57	Vitamins	BNF 9.6
58	Drugs used in rheumatic diseases and gout	BNF 10.1
59	Drugs for soft-tissue disorders and topical pain relief	BNF 10.3
60	Anti-infective eye preparations	BNF 11.3
61	Miscellaneous ophthalmic preparations	BNF 11.8
62	Emollient and barrier preparations	BNF 13.2
63	Topical local anesthetics and antipruritic	BNF 13.3
64	Topical corticosteroids	BNF 13.4
65	Anti-infective skin preparations	BNF 13.10
Lab values (latest record within 5 years before index date)		
66	ALT	Alanine aminotransferase (U/L) (continuous)
67	Albumin	Albumin (g/L) (continuous)
68	ALP	Alkaline phosphatase (U/L) (continuous)
69	Bilirubin	Bilirubin (umol/L) (continuous)
70	Calcium	Calcium (mmol/L) (continuous)
71	Creatinine	Creatinine (umol/L) (continuous)
72	HCT	Hematocrit (L/L) (continuous)

Supplementary Table 1. *(continued)*

No.	Variable	Description
73	MCHC	Mean corpuscular hemoglobin concentration (g/dL) (continuous)
74	MCV	Mean corpuscular volume (fL) (continuous)
75	MPV	Mean platelet volume (fL) (continuous)
76	Phosphate	Phosphate (mmol/L) (continuous)
77	Potassium	Potassium (mmol/L) (continuous)
78	Protein	Protein (g/L) (continuous)
79	RDW	Red cell distribution width (%) (continuous)
80	Sodium	Sodium (mmol/L) (continuous)
81	Urea	Urea (mmol/L) (continuous)
82	WBC	White blood cell count (x10^9/L) (continuous)

Supplementary Table 2. Final hyperparameters used in the machine learning models.

Outcome	Model	Method[a]	Hyperparameters[b]
**1-year mortality**	Stepwise LR	glmStepAIC	N/A
	Neural network	nnet	size=3, decay=0.5
	Random forest	ranger	mtry=2, splitrule=1, min.node.size=20
	Gradient boosting machine	gbm	n.trees=200, interaction.depth=1, shrinkage=0.1, n.minobsinnode=41
	XGBoost	xgbTree	nrounds=150, max_depth=1, eta=0.3, gamma=0, colsample_bytree=0.6, min_child_weight=1, subsample=1
**2-year mortality**	Stepwise LR	glmStepAIC	N/A
	Neural network	nnet	size=3, decay=0.5
	Random forest	ranger	mtry=2, splitrule=1, min.node.size=10
	Gradient boosting machine	gbm	n.trees=300, interaction.depth=1, shrinkage=0.1, n.minobsinnode=31
	XGBoost	xgbTree	nrounds=150, max_depth=1, eta=0.3, gamma=0, colsample_bytree=0.8, min_child_weight=1, subsample=1
**5-year mortality**	Stepwise LR	glmStepAIC	N/A
	Neural network	nnet	size=3, decay=0.5
	Random forest	ranger	mtry=2, splitrule=1, min.node.size=20
	Gradient boosting machine	gbm	n.trees=250, interaction.depth=1, shrinkage=0.1, n.minobsinnode=41
	XGBoost	xgbTree	nrounds=150, max_depth=1, eta=0.3, gamma=0, colsample_bytree=0.6, min_child_weight=1, subsample=1

LR, logistic regression; *XGBoost*, eXtreme Gradient Boosting.

[a] The methods used in training the models in the R package “caret”. Example codes are provided in Appendix.

[b] Hyperparameters were tuned using grid search with 10 rounds of 10-fold cross-validation.

Supplementary Table 3. Characteristics by death status at 1-year after age 100.

Characteristic	Alive at 1-year (n=7,226)	Died at 1-year (n=2,492)	*p*[a]
**Demographics, n (%)**			
Male sex	1166 (16.1)	483 (19.4)	<0.001
Number of hospitalizations in past year	1.04 (1.67)	2.13 (2.76)	<0.001
**Presence of disease diagnoses, n (%)**			
Alcohol misuse	727 (10.1)	289 (11.6)	0.034
Atrial fibrillation	656 (9.1)	359 (14.4)	<0.001
Bacterial infection	2276 (31.5)	1186 (47.6)	<0.001
Chronic obstructive pulmonary disease (COPD)	649 (9.0)	340 (13.6)	<0.001
Cataract	746 (10.3)	210 (8.4)	0.007
Coronary heart disease	478 (6.6)	234 (9.4)	<0.001
Delirium	338 (4.7)	164 (6.6)	<0.001
Dementia	1488 (20.6)	790 (31.7)	<0.001
Diabetes	488 (6.8)	221 (8.9)	0.001
Infection - ear/upper respiratory tract	627 (8.7)	250 (10.0)	0.046
Hip fracture	979 (13.5)	350 (14.0)	0.556
Gout	514 (7.1)	200 (8.0)	0.144
Heart failure	923 (12.8)	519 (20.8)	<0.001
Hypertension	2452 (33.9)	1020 (40.9)	<0.001
Infection - skin	616 (8.5)	332 (13.3)	<0.001
Infection - other organs	881 (12.2)	479 (19.2)	<0.001
Infection - digestive system	730 (10.1)	371 (14.9)	<0.001
Intracerebral hemorrhage	701 (9.7)	351 (14.1)	<0.001
Ischemic stroke	932 (12.9)	447 (17.9)	<0.001
Lower respiratory tract infection	2187 (30.3)	1187 (47.6)	<0.001
Osteoarthritis	455 (6.3)	154 (6.2)	0.873
Other anemia	783 (10.8)	480 (19.3)	<0.001
Infection - other organisms	3078 (42.6)	1474 (59.1)	<0.001
Sepsis	712 (9.9)	422 (16.9)	<0.001
Stable angina	583 (8.1)	244 (9.8)	0.009
Subarachnoid hemorrhage	676 (9.4)	345 (13.8)	<0.001
Transient ischemic attack (TIA)	350 (4.8)	151 (6.1)	0.021
Urinary tract infection (UTI)	1634 (22.6)	886 (35.6)	<0.001
Viral infection	775 (10.7)	396 (15.9)	<0.001
**Drug prescriptions, n (%)**			
Dyspepsia and gastro-oesophageal reflux disease	1044 (14.4)	307 (12.3)	0.009
Antisecretory drugs and mucosal protectants	2935 (40.6)	1316 (52.8)	<0.001
Laxatives	3358 (46.5)	1403 (56.3)	<0.001
Diuretics	1458 (20.2)	781 (31.3)	<0.001
Beta-adrenoceptor blocking drugs	893 (12.4)	343 (13.8)	0.075
Hypertension and heart failure	1647 (22.8)	667 (26.8)	<0.001
Nitrates, calcium-channel blockers & other antianginal drugs	3333 (46.1)	1206 (48.4)	0.053
Antiplatelet drugs	1777 (24.6)	726 (29.1)	<0.001
Lipid-regulating drugs	597 (8.3)	179 (7.2)	0.095
Bronchodilators	888 (12.3)	522 (20.9)	<0.001
Antihistamines, hypo-sensitization and allergic emergencies	1634 (22.6)	629 (25.2)	0.008
Mucolytics	1549 (21.4)	896 (36.0)	<0.001
Cough preparations	2007 (27.8)	839 (33.7)	<0.001
Hypnotics and anxiolytics	443 (6.1)	222 (8.9)	<0.001
Drugs used in psychoses and related disorders	718 (9.9)	339 (13.6)	<0.001

Supplementary Table 3. *(continued)*

Characteristic	Alive at 1-year (n=7,226)	Died at 1-year (n=2,492)	p[a]
Antidepressant drugs	523 (7.2)	200 (8.0)	0.212
Drugs used in nausea and vertigo	936 (13.0)	376 (15.1)	0.008
Analgesics	4049 (56.0)	1555 (62.4)	<0.001
Antibacterial drugs	2896 (40.1)	1517 (60.9)	<0.001
Drugs used in diabetes	404 (5.6)	184 (7.4)	0.001
Corticosteroids (endocrine)	357 (4.9)	252 (10.1)	<0.001
Drugs for genito-urinary disorders	393 (5.4)	190 (7.6)	<0.001
Anemias and some other blood disorders	970 (13.4)	495 (19.9)	<0.001
Fluids and electrolytes	1535 (21.2)	924 (37.1)	<0.001
Minerals	853 (11.8)	325 (13.0)	0.11
Vitamins	1870 (25.9)	827 (33.2)	<0.001
Drugs used in rheumatic diseases and gout	884 (12.2)	304 (12.2)	0.992
Drugs for soft-tissue disorders and topical pain relief	1968 (27.2)	522 (20.9)	<0.001
Anti-infective eye preparations	400 (5.5)	167 (6.7)	0.036
Miscellaneous ophthalmic preparations	1979 (27.4)	607 (24.4)	0.003
Emollient and barrier preparations	2449 (33.9)	1058 (42.5)	<0.001
Topical local anesthetics and antipruritics	679 (9.4)	284 (11.4)	0.004
Topical corticosteroids	1155 (16.0)	456 (18.3)	0.008
Anti-infective skin preparations	931 (12.9)	507 (20.3)	<0.001
**Lab values, mean (SD)**			
Alanine aminotransferase (U/L)	15.18 (13.73)	17.02 (21.69)	<0.001
Albumin (g/L)	34.33 (4.63)	31.90 (5.41)	<0.001
Alkaline phosphatase (U/L)	84.50 (34.39)	89.68 (47.37)	<0.001
Bilirubin (umol/L)	10.70 (5.56)	10.68 (6.66)	0.88
Calcium (mmol/L)	2.20 (0.11)	2.17 (0.13)	<0.001
Creatinine (umol/L)	91.83 (35.79)	99.46 (61.01)	<0.001
Hematocrit (L/L)	0.34 (0.04)	0.33 (0.05)	<0.001
Mean corpuscular hemoglobin concentration (g/dL)	33.36 (0.90)	33.28 (1.06)	0.001
Mean corpuscular volume (fL)	90.86 (7.07)	90.95 (7.72)	0.586
Mean platelet volume (fL)	8.72 (1.10)	8.65 (1.21)	0.011
Phosphate (mmol/L)	1.06 (0.18)	1.09 (0.22)	<0.001
Potassium (mmol/L)	4.05 (0.46)	4.12 (0.55)	<0.001
Protein (g/L)	68.45 (6.40)	67.30 (7.57)	<0.001
Red cell distribution width (%)	14.31 (1.55)	14.82 (2.05)	<0.001
Sodium (mmol/L)	138.83 (3.52)	138.13 (4.51)	<0.001
Urea (mmol/L)	7.34 (3.09)	8.61 (5.20)	<0.001
White blood cell count (x10^9/L)	7.62 (2.92)	8.18 (3.50)	<0.001

[a] *P*-values were based on *t*-tests for continuous variables and χ^2 tests for categorical variables.

Supplementary Table 4. Model performance in predicting 5-year mortality.

Model performance	Stepwise LR	Neural network	Random forest	Gradient boosting machine	XGBoost
Training cohort					
AUROC (95% CI)	0.691 (0.678–0.703)	0.736 (0.721–0.751)	0.973 (0.970–0.976)	0.751 (0.736–0.765)	0.757 (0.742–0.771)
Testing cohort					
AUROC (95% CI)	0.703 (0.684–0.722)	0.697 (0.675–0.720)	0.690 (0.667–0.713)	0.698 (0.675–0.721)	0.700 (0.678–0.723)
P-value (DeLong)	Reference	0.467	0.19	0.826	0.848
Brier score	0.266	0.141	0.141	0.141	0.140
Sensitivity	0.553	0.444	0.000	0.333	0.286
Specificity	0.608	0.816	0.815	0.816	0.816
PPV	0.018	0.007	0.000	0.007	0.007
NPV	0.990	0.998	1.000	0.997	0.996
F1 score	0.035	0.015	N/A	0.015	0.015
Accuracy	0.607	0.815	0.815	0.814	0.813

AUROC, area under the receiver operating characteristic curve; *CI*, confidence interval; *LR*, logistic regression; *NPV*: negative predictive value; *PPV*: positive predictive value; *XGBoost*, eXtreme Gradient Boosting.

Supplementary Table 5. Model performance in the sensitivity analysis limiting to individuals died at age <110 years (n=2,831).

Model performance	Stepwise LR	Neural network	Random forest	Gradient boosting machine	XGBoost
**1-year mortality**					
AUROC (95% CI)	0.698 (0.675–0.722)	0.696 (0.672–0.719)	0.702 (0.679–0.725)	0.700 (0.678–0.723)	0.702 (0.679–0.725)
P-value (DeLong)	Reference	0.329	0.533	0.638	0.338
Brier score	0.167	0.168	0.168	0.167	0.167
Sensitivity	0.790	0.787	0.774	0.787	0.788
Specificity	0.597	0.578	0.629	0.589	0.594
PPV	0.944	0.943	0.970	0.946	0.946
NPV	0.247	0.236	0.151	0.233	0.236
F1 score	0.860	0.858	0.861	0.859	0.860
Accuracy	0.770	0.766	0.765	0.767	0.768
**2-year mortality**					
AUROC (95% CI)	0.690 (0.671–0.710)	0.691 (0.672–0.711)	0.696 (0.677–0.715)	0.698 (0.678–0.717)	0.698 (0.679–0.718)
P-value (DeLong)	Reference	0.872	0.283	0.074	0.050
Brier score	0.220	0.221	0.221	0.218	0.218
Sensitivity	0.639	0.644	0.640	0.644	0.643
Specificity	0.657	0.667	0.647	0.655	0.657
PPV	0.772	0.779	0.754	0.761	0.765
NPV	0.501	0.507	0.516	0.519	0.515
F1 score	0.699	0.705	0.693	0.698	0.699
Accuracy	0.645	0.652	0.643	0.648	0.648
**5-year mortality**					
AUROC (95% CI)	0.697 (0.677–0.716)	0.689 (0.665–0.713)	0.686 (0.661–0.711)	0.693 (0.669–0.718)	0.695 (0.670–0.719)
P-value (DeLong)	Reference	0.501	0.521	0.724	0.517
Brier score	0.258	0.128	0.127	0.128	0.127
Sensitivity	0.500	0.375	0.000	0.273	0.167
Specificity	0.625	0.840	0.840	0.840	0.840
PPV	0.016	0.007	0.000	0.007	0.004
NPV	0.990	0.998	1.000	0.997	0.996
F1 score	0.031	0.013	N/A	0.013	0.009
Accuracy	0.624	0.839	0.839	0.838	0.837

AUROC, area under the receiver operating characteristic curve; *CI*, confidence interval; *LR*, logistic regression; *NPV*: negative predictive value; *PPV*: positive predictive value; *XGBoost*, eXtreme Gradient Boosting.

Supplementary Table 6. Characteristics of the independent cohort of oldest-old adults.

Characteristic	Age groups				
	85–89 years	90–94 years	95–99 years	100–105 years	*p*
Number of individuals (%)[a]	106,270 (60.9)	50,154 (28.7)	15,326 (8.8)	2,856 (1.6)	
Sex, n (%)					<.001
Women	62,234 (58.6)	33,385 (66.6)	11,286 (73.6)	2,329 (81.5)	
Men	44,036 (41.4)	16,769 (33.4)	4,040 (26.4)	527 (18.5)	
1-year mortality, n (%)	8,402 (7.9)	6,661 (13.3)	3,121 (20.4)	795 (27.8)	<.001
2-year mortality, n (%)	17,012 (16.0)	12,898 (25.7)	5,732 (37.4)	1,399 (49.0)	<.001
5-year mortality, n (%)	44,720 (42.1)	29,901 (59.6)	11,688 (76.3)	2,481 (86.9)	<.001
Main cause of death, n (%)					<.001
Malignant neoplasms	5,904 (5.6)	2,473 (4.9)	568 (3.7)	59 (2.1)	
Pneumonia	19,537 (18.4)	14,252 (28.4)	5,921 (38.6)	1,240 (43.4)	
Diseases of heart	5,966 (5.6)	3,701 (7.4)	1,234 (8.1)	214 (7.5)	
Cerebrovascular diseases	1,779 (1.7)	1,096 (2.2)	350 (2.3)	71 (2.5)	
Nephritis, nephrotic syndrome and nephrosis	2,026 (1.9)	1,122 (2.2)	323 (2.1)	46 (1.6)	
Dementia	467 (0.4)	497 (1.0)	293 (1.9)	83 (2.9)	
Septicaemia	1,504 (1.4)	915 (1.8)	354 (2.3)	86 (3.0)	
Chronic lower respiratory diseases	571 (0.5)	242 (0.5)	74 (0.5)	12 (0.4)	
Diabetes	115 (0.1)	55 (0.1)	25 (0.2)	1 (0.0)	
COVID-19	1,346 (1.3)	834 (1.7)	295 (1.9)	64 (2.2)	
All other causes	8,916 (8.4)	6,100 (12.2)	2,350 (15.3)	528 (18.5)	
Unknown	3,669 (3.5)	2,365 (4.7)	852 (5.6)	191 (6.7)	
Alive during 5-year follow-up	54,470 (51.3)	16,502 (32.9)	2,687 (17.5)	261 (9.1)	
Albumin, g/L, mean (SD)	37.83 (5.28)	36.34 (5.49)	34.73 (5.56)	33.49 (5.60)	<.001
Urea, mmol/L, mean (SD)	6.94 (3.49)	7.18 (3.68)	7.35 (3.69)	7.56 (3.81)	<.001
Number of hospitalizations in past year, mean (SD)	0.98 (2.46)	1.13 (2.18)	1.27 (2.22)	1.39 (2.20)	<.001
HFRS, mean (SD)	4.20 (5.71)	5.71 (6.57)	7.03 (7.05)	7.86 (7.22)	<.001
CCI, mean (SD)	0.60 (1.20)	0.72 (1.26)	0.78 (1.30)	0.78 (1.26)	<.001

CCI, Charlson comorbidity index; *HFRS*, Hospital Frailty Risk Score; *SD*, standard deviation.

[a] The cohort included all oldest-old adults aged 85–105 years in 2019 identified from the CDARS (n=174,606). Index date was defined as January 1, 2019.

Appendix. R codes for development of machine learning models

```
library(caret)
library(pROC)
library(rms)

## train ----
# control parameters
ctrl <- trainControl(
  method = "repeatedcv",
  number = 10,
  repeats = 10,
  classProbs = TRUE,
  summaryFunction = twoClassSummary
);

fits <- train(
  as.formula(paste(dep_col, "~ .")),
  data = train_df,
  method = "ranger",  # glmStepAIC, nnet, ranger, gbm, or xgbTree
  metric = "ROC",
  trControl = ctrl
);

pred_prob_train <- predict(fits, train_df, type = "prob");

# confusion matrix
conf_train <- confusionMatrix(pred_classes_train, train_df[, dep_col]);

# area under the curve
auc_train <- pROC::auc(train_df[, dep_col], pred_prob_train$yes); print(auc_train)
ci_auc_train <- ci.auc(train_df[, dep_col], pred_prob_train$yes); print(ci_auc_train)

# calibration
val.prob(pred_prob_train$yes, as.numeric(test_df[, dep_col])-1, statloc = FALSE)
```
